# The antifibrotic effect of isolate tagitinin C from tithonia diversifolia (Hemsley) A. Gray on keloid fibroblast cell

**DOI:** 10.11604/pamj.2018.30.264.9994

**Published:** 2018-08-08

**Authors:** Imaniar Ranti, Mae Sri Hartati Wahyuningsih, Yohannes Widodo Wirohadidjojo

**Affiliations:** 1 Department of Pharmacology, Faculty of Medicine and Health Science,Universitas Muhammadiyah Yogyakarta, Yogyakarta, Indonesia; 2 Department of Pharmacology, Faculty of Medicine, Universitas Gadjah Mada, Yogyakarta, Indonesia; 3 Department of Dermatology and Venerology, Faculty of Medicine, Universitas Gadjah Mada, Yogyakarta, Indonesia

**Keywords:** Cell proliferation, collagen deposition, cytoxicity study, keloid, taginin C

## Abstract

**Introduction:**

Keloids characterized by fibroblast hyperproliferation and depositions of collagen which similar to cancer cells. Tagitinin C is a class of sesquiterpene lactones (SLS) was isolated from the leaves of the moon flower *Tithonia diversifolia* (Hemsley) A. Gray. The study aim is to evaluate the effects of tagitinin C from *Tithonia diversifolia* to keloid fibroblasts (KF).

**Methods:**

Monolayer cultures of keloid fibroblast (three passages) were treated with 8 serial concentration of tagitinin C (0.015 to 2) μg/mL during 72 and 120 hours. A positive control using mitomycin C. Cellular viabilities were measured by MTT assay. Collagen depositions were measured by Sirius Red assay for nonsoluble collagen.

**Results:**

The reading of the result was conducted by ELISA reader. Data were analyzed by probit regression with SPSS 19 for Windows. The result showed that tagitinin C can inhibit keloid fibroblasts (KF) viability with IC_50_ 0.122 μg/mL (incubation 72h) and 0.039 μg/mL (120h), whereas mitomycin C IC_50_ 0.120 μg/mL (72h) and IC_50_ of 0.100 μg/mL (120h). At IC_50_ concentration of tagitinin C on keloid collagen deposition 53.1% (72h) and 44.3% (120h), whereas the IC_50_ concentration of mitomycin C on keloid collagen deposition 60.4% (72h) and 52.1% (120h). Selectivity index tagitinin C on normal fibroblasts (NF) is 287 for 72h incubation and 791 for 120h incubation.

**Conclusion:**

It can be concluded that the ability of tagitinin C inhibits KF viability and decreasing keloid collagen deposition is consistent with the concentration (concentration-dependent) and incubation time (time-dependent). Tagitinin C has a low toxicity level on NF with high selectivity index.

## Introduction

Keloids are fibroproliferative benign tumors on the dermis layer of skin. It is caused by an excessive wound healing response [1]. In developing countries, there are around 100 million patients who have scarring complaints. Those are giving the impact on physical condition, aesthetic, psychological, and social [2,3]. Therefore, the research to looking for an antifibrotic agent keloids need to be developed. Keloids characterized by fibroblast hyper proliferations and depositions of collagen from fibroblast. The characteristic of fibroblast keloid is similar to the cancer cell characteristic [4]. Some of them are able to provide for their own growth signals, able to synthesize growth factor, able to replicate continuously, able to initiate angiogenesis signaling, and loss of ability to apoptosis. The difference is keloid fibroblasts unable to metastasis like cancer cell [5]. Tagitinin C is the class of *sesquiterpene lactones* (SLs) compounds, which is isolated from (*Tithonia diversifolia*(Hemsley) A. Gray) leaf using Bioassay-Guided Isolation methods (MTT pada sel HeLa IC_50_: 9,776μg/mL) [6]. The previous study has done the cytotoxic test from pure isolate of *Tithonia diversifolia* against various human cancer cells and normal cells in vitro. It was done to determine its selectivity [7]. The previous research has shown that tagitinin C from *Tithonia diversifolia* tagitinin is the most active and the most selective in colon cancer cells (WiDr) with the selectivity index around 69.015. In addition, melanoma cells or skin cancer cells (M19) have the selectivity index around 40.536 (IC_50_ = 0,996μg/mL) [7,8]. The research on the antifibrotic effects of isolates tagitinin C from *Tithonia diversifolia* against keloid fibroblast cell has never been studied previously. Therefore, this research is conducted to examine the antifibrotic effects of isolates tagitinin C from *Tithonia diversifolia* against keloid fibroblasts which is measured from cell proliferation, deposition of collagen keloids, and toxicity tests of tagitinin C toward normal fibroblasts.

## Methods

**The subculture of keloids fibroblast:** Subculturing was done by discarding medium in the flash. It was washed by 10 cc sterile PBS (Phosphate Buffer Saline). Then, fibroblasts harvested using warm trypsinization techniques. Trypsinization conducted by using trypsin 0.25% 2 cc and incubated for 2-3 minutes until all fibroblasts regardless from the basis flask. Trypsin was neutralized by adding complete DMEM medium until 3 times the volume of trypsin. The cell suspension in the flask was transferred into 15 cc tube using a Pasteur pipette, it was conducted by vortex then centrifuged with the a speed of 200 MG for 10 min, the supernatant was discarded carefully, cell was washed by 8 cc Sodium Chloride and it was conducted by vortex, centrifuged with the 200g for 10 minutes and the supernatant was discarded carefully. The pellet was added with 1 cc of complete DMEM medium and conducted by vortex in order pellets and medium were mixed equitably. The mixture was divided into several sterile flasks and cultured in an incubator with 5% CO2 concentration, temperature 37^°^C, for 24 hours. The medium was changed every 3 days, until the cells fulfilling 50% on surface area flask, and can be harvested. The whole process of making the subculture could be repeated to obtain a subculture passage 3, after that the cells were harvested in the same way.

**The series preparation of material concentration test:** The isolate of tagitinin C was weighed 1.6 mg, dissolved with the 160 μL DMSO to obtain the concentration of stock solution 10.000 μg/mL, the 8 series concentration were made with the different concentration (0.015; 0.031; 0.062; 0.125; 0.25; 0.5; 1; 2) μg/mL. Mitomycin C-Kyowa^®^ (2mg/5ml) was made serial concentration in the same way like tagitinin C.

**The treatment of keloid fibroblast cell:** This study was conducted by using four pieces of microplate. Each microplate contains 96 wells. Every well filled by 200 μL of complete DMEM medium containing keloid fibroblasts with the number of density 5 x 10^3^ cells/well. Then it was incubated for 24 hours. The next day the media was discarded and changed with the series concentration from material test on complete DMEM medium. Every concentration was made triplicate. The cell cultures were incubated for 72 hours and 120 hours at 37^°^C, 5% CO_2_. Toxicity test was done by using 2 microplates within 96 wells containing normal fibroblasts.

**The measurement of keloid fibroblast proliferation by MTT method:** The investigation of keloid fibroblast proliferation was conducted by MTT method. This method was begun by sucking the entire existing medium in each well. Then inserted 200 μL new complete media and added 50 μL MTT solution with 5mg/mL concentration in each well. Furthermore, the plate was wrapped in aluminum foil and then incubated in CO_2_incubator with a temperature of 37^°^C for 6 hours. Medium and MTT solution in the wells was removed, replaced by 200 μL DMSO on each well then shaken. Furthermore, the absorbance was read by using multiplates reader at a wavelength of 570 nm. Toxicity test on normal fibroblasts was conducted by using MTT method.

**Isolation of *Tagitinin C* from *T. diversifolia* (Hemsley) A. Gray Leaves:** *Tagitinin C* from *T. diversifolia* leaves was isolated from the chloroform extract in laboratory of Pharmacology and Therapy, Faculty of Medicine, Universitas Gadjah Mada, Yogyakarta, Indonesia. The isolation of *Tagitinin C* from *T. diversifolia* was done according to Bioassay Guided Isolation method. Each extract, fractions, or compounds obtained were monitored by cytotoxic assay. *Tagitinin C* was identified according its spectroscopic (UV, IR, ^13^C- and 1H-NMR) data used for further investigation.

**The measurement of keloid collagen deposition by Sirius Red method:** Media from 96 wells were sucked then those were washed with 200 μL PBS in each well 3 times. Fixation was conducted by Bouin solution for 1 hour, washed with inserted into a plastic which contained water until microplate submerged so that the yellow color disappear. Furthermore, the microplate was drained on wipes at room temperature overnight. Then, each of wells was added 200 μL sirius red which has been diluted by saturated picric acid, incubated for 1 hour, then removed sirius red which was not bound and washed by HCL 200 μL 0,1N 3 times until sirius red color clean from the walls and the supernatant. After that, 200 μL 0,5N NaOH was added and waited for 30 minutes. Absorbance was read at a wavelength of 570 nm by multiplates reader.

**Data analysis:** The mean was recorded and counted from the measurement results that were obtained from the *multiplate reader*. Based on those calculations, mean of optical density from the negative control group, positive control group, and therapy group was obtained with various series of concentration, and thus, the percentage of cell viability could be calculated by the following formula: After the percentage of cell viability on each series of concentration was obtained, then the concentration of concentration series from isolates that could inhibit the growth of 50% cell population (IC_50_) was calculated with probit regression analysis by using SPSS version 19 for Windows.

## Results

**Figure 1:**
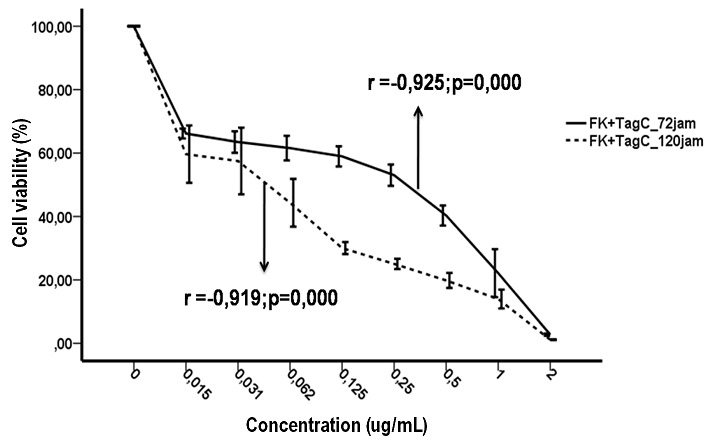
Correlation between concentration of tagitinin C isolates from *T. diversifolia* on the keloid fibroblasts cell viability with 72 hours and 120 hours incubation times

**Fibroblasts cell proliferation:** A decreased amount of KF cell viability was found after the administration of tagitinin C isolate. The decrease in KF cell viability was in accordance with the concentration increase in tagitinin C isolates from *Tithonia diversifolia* with a strong correlation value (r = -0.924; p = 0.000 for 72 hours incubation; r = -0.919; p = 0.000 for 120 hours incubation) (Figure 1). Antifibrotic activity of tagitinin C isolates from *Tithonia diversifolia* and mitomycin C on KF cell proliferation was expressed in IC_50_ values. From the data above, the IC_50_ values were as follows. Results of independent t-test showed that IC_50_ value of tagitinin C between 72 hours and 120 hours incubations was significantly different (p < 0.05), which means that the longer the incubation time, the lower concentration of tagitinin C was needed to inhibit cell proliferation by 50% on KF cells population by in vitro, whereas the IC_50_ value of mitomycin C between 72 hours and 120 hours incubation was not significantly different (Table 1).

**Table 1:** IC_50_ value of tagitinin C isolates from *T. diversifolia* andmitomycin C in keloid fibroblasts cells

Material	IC_50_ Value (μg/mL)		
	72 hours	120 hours	*P*
Tagitinin C	0,122 ± 0,026	0,039 ± 0,028	0,024
Mitomycin C	0,120 ± 0,034	0,100 ± 0,051	0,621

Antifibrotic effect of tagitinin C isolates from *T. diversifolia* on keloid collagen deposition

Result of keloid collagen deposition on tagitinin C IC_50_ concentration were 0.122 mg/mL (72 hours) and 0.039 mg/mL (120 hours), and mitomycin C IC_50_ were 0.120 mg/mL (72 hours) and 0.100 mg/mL (120 hours), presented in Table 2 as follows. Independent t-test results showed that keloid collagen deposition of tagitinin C and mitomycin C between 72 hours and 120 hours incubations was significantly different (p < 0.05), which means that the longer the incubation time, the fewer keloid collagen deposition, but the keloid collagen deposition that was given tagitinin C treatment was fewer than mitomycin C (Table 2).

**Table 2:** Average of keloid collagen deposition after the administration tagitinin C isolates from *T. diversifolia* andmitomycin C in keloid fibroblast cells

Material	percentage of keloidcollagen deposition(Mean±SD)		
	72 jam	120 jam	p
Tagitinin C	53,1 ± 2,39	44,3 ± 1,04	0,004
Mitomycin C	60,4 ± 0,85	52,1 ± 2,38	0,005

**Figure 2:**
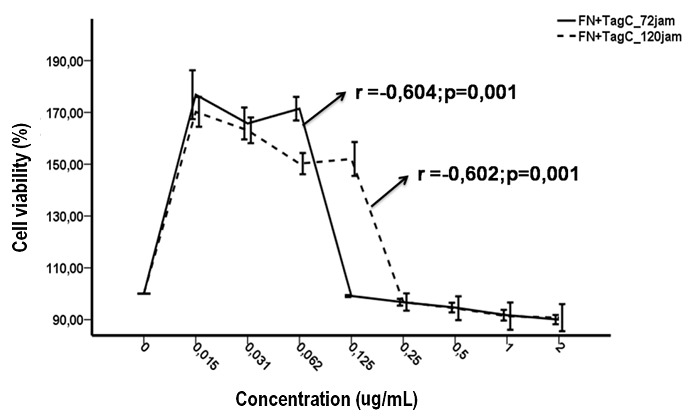
Correlation between concentration of tagitinin C isolates from *T. diversifolia* on the cells viability of normal fibroblast with 72 hours and 120 hours incubation times

**Toxicity test on Tagitinin C isolates of *T. diversifolia* against the normal fibroblast cell:** Based on the toxicity test, tagitinin C of *T. diversifolia* against normal fibroblast cells showed that the tagitinin C isolates of *T. diversifolia* at less than 0.125 mg/mL concentration actually increased the NF cell viability, although after 0.125 mg/mL for 72 hours incubation period and 0.25 mg/mL for 120 hours incubation period showed no difference in NF cell viability against the negative control (Anova: LSD: p > 0.05 were not written in the chart) (Figure 2). Toxicity test on normal fibroblasts was performed to determine the selectivity of the test compounds. Based on cell viability data of the normal fibroblast percentage on various concentration of the test substances, the IC_50_values of tagitinin C isolates from *T. diversifolia* and mitomycin C in normal fibroblast cell was obtained. IC_50_value ratio in normal fibroblast cells to the IC_50_ values in keloid fibroblasts cells was calculated to determine the selectivity of the test material (Table 3).

**Table 3:** IC_50_ value of tagitinin C isolates from *T. diversifolia* and mitomycin C innormal fibroblast cells

Indicator	Material			
	Tagitinin C		Mitomycin C	
	72 hours	120 hours	72 hours	120 hours
IC_50_ on NF (μg/mL)	35,02	30,85	16,21	13,89
IC_50_ on KF (μg/mL)	0,122	0,039	0,120	0,100
Index of selectivity	287	791	135	138

## Discussion

Cell proliferation is a physiological process of cell growth that is followed by cell division. Cell proliferation ability is balanced with the programmed cell death ability (apoptosis) in normal circumstances, resulting in a balance in the maintenance of tissues and organs integrity (homeostasis) [8]. On keloid, the regulatory function failed, resulting in an increase of fibroblast cell proliferation and a decrease in cell mortality [9]. From various studies that evaluated the effect of tagitinin C anticancer isolates, it can be presumed that tagitinin C isolates have the cytotoxic ability through various channels, for example, tagitinin C isolates can increase the expression of p53 protein in HeLa cells, thereby, can enhance the apoptosis ability [10]. Another study stated that tagitinin C isolates can reduce VEGF expression in colon cancer cells (WiDr) [11]. The VEGF expression inhibition mechanism by tagitinin C isolates is suspected to be caused by NF-kB transcription barriers through the alkylation on cystine residue (Cys 38) p56 [12, 13]. This study also reported that that the longer the incubation time, the fewer keloid collagen deposition, but the keloid collagen deposition with tagitinin C treatment was fewer than treatment with mitomycin C. The collagen synthesis process began when the fibroblast got a stimulus from TGF-β that was bound to TGF-β receptors on the fibroblasts membrane. TGF-β normally stored in an inactive state and bound to a particular latent protein. In time of injury, the TGF-β will be separated from the binding protein and will be activated to respond to mechanical trauma that occurs [14]. In normal circumstances, the TGF-β signaling is regulated by *peroxisome proliferator-activated receptor gamma* (PPAR-γ), which is one of the transcription factors on nuclear receptor that inhibits the fibrogenic excessive response [15]. Class of *sesquiterpene lactones* from *T. diversifolia* is known as PPAR α/γ dual agonist that includes tirotundin and tagitinin A [16]. Further research to examine the inhibition mechanism of keloid collagen deposition by tagitinin C isolates from *T. diversifolia* through PPARs is very interesting to be done.

Based on the toxicity test, tagitinin C of *T. diversifolia* increased the NF cell viability. Previous research showed that basal level of NF-κB in keloid fibroblast is higher than normal fibroblast. It explained that NF-_κ_B also plays a role in the keloid pathogenesis, especially in the collagen synthesis of keloid [17]. Research on *Xanthium stramarium* (XAS) and *Psoralea corylifolia* (PSC), that is a compound of *sesquiterpene lactones*, is known to inhibit the keloid fibroblasts proliferation, inhibit TGF-β1, and inhibit collagen synthesis of keloid through the NF-κB [18]. Further research to look at the inhibition mechanism of keloid collagen deposition by tagitinin C isolates from *T. diversifolia* through inhibition of NF-κB is also very interesting to be done. The compound that is preferred for the development of new drugs is a selective compound. The means of selectivity is the selectivity in inhibiting the biological process that is only related to abnormal cell/tissue, without affecting normal cell/tissue. In this research, results showed that tagitinin C isolates from *T. diversifolia* has high selectivity to normal fibroblasts. According to Janett-Siems et al. (1999), index of selectivity at above 10 indicates that the substance is selective, so the higher the selectivity index, the better the compound is [19]. According to previous study, there are several levels of toxicity based on the number of cell viability compared to control group, that is: (1) low toxicity if cell viability is between 60-90%; (2) moderate toxicity if cell viability is between 30-59%; and (3) high toxicity if cell viability is < 30% [19]. Based on the calculation results on the cell viability percentage of normal fibroblast, it can be concluded that the tagitinin C isolates of *T. diversifolia* and mitomycin C were included in the low toxicity criteria in NF cells.

## Conclusion

In the 72 hours incubation, IC _50_ value of tagitinin C was 0.122 mg/mL with a keloid collagen deposition was at 53.1%, while in the 120 hours incubation, IC_50_value of tagitinin C was 0.039 mg/mL with keloid collagen deposition was at 44.3%. Based on the Sjogren *et al.* (2000) criteria, tagitinin C of *T. diversifolia* was classified into low toxicity category against the NF cells with a high index of selectivity. In summary, tagitinin C has a potential activity to inhibit keloid fibroblast viability and to decrease keloid collagen deposition. This study proved a novel evidence that tagitinin C is non-toxic and selective for anti-fibrotic agent.

### What is known about this topic

- Fibroblast hyperproliferation and depositions of collagen;
- Tagitinin C's effects to Keloid Fibroblast.

### What this study adds

- The effects of tagitinin C from *T. diversifolia* to keloid fibroblasts (KF);
- The ability of tagitinin C inhibits KF viability and decreasing keloid collagen deposition is consistent with the concentration (concentration-dependent) and incubation time (time-dependent);
- Tagitinin C has a low toxicity level on NF with high selectivity index.

## Competing interests

The authors declare no competing interests.
